# Complete Genome Sequence of *Serratia marcescens* WW4

**DOI:** 10.1128/genomeA.00126-13

**Published:** 2013-04-04

**Authors:** Wan-Chia Chung, Ling-Ling Chen, Wen-Sui Lo, Pei-An Kuo, Jenn Tu, Chih-Horng Kuo

**Affiliations:** a Institute of Plant and Microbial Biology, Academia Sinica, Taipei, Taiwan; b Molecular and Biological Agricultural Sciences Program, Taiwan International Graduate Program, National Chung Hsing University and Academia Sinica, Taipei, Taiwan; c Graduate Institute of Biotechnology, National Chung Hsing University, Taichung, Taiwan; d Institute of Biotechnology, National Tsing Hua University, Hsinchu, Taiwan; e Biotechnology Center, National Chung Hsing University, Taichung, Taiwan

## Abstract

*Serratia marcescens* WW4 is a biofilm-forming bacterium isolated from paper machine aggregates. Under conditions of phosphate limitation, this bacterium exhibits intergeneric inhibition of *Pseudomonas aeruginosa*. Here, the complete genome sequence of *S. marcescens* WW4, which consists of one circular chromosome (5,241,455 bp) and one plasmid (pSmWW4; 3,248 bp), was determined.

## GENOME ANNOUNCEMENT

The bacterial strain *Serratia marcescens* WW4 was isolated from a paper machine in Taiwan (1). Phenotypic characterization of this bacterium revealed that it is capable of forming biofilms individually or with *Pseudomonas aeruginosa*. Intriguingly, while the two bacteria may coexist in LB medium or M9 minimal medium, phosphate limitation can induce intergeneric inhibition of *P. aeruginosa* by *S. marcescens* WW4 (1).

To determine the complete genome sequence of *S. marcescens* WW4, we performed whole-genome shotgun sequencing using one Illumina paired-end library with an average insert size of ~325 bp. With the 150-bp paired-end reads generated using the Genome Analyzer IIx platform (Illumina), we obtained ~8 Gb of raw reads. These reads were trimmed using a quality score cutoff of 20 and a length cutoff of 70 bp. The initial *de novo* genome assembly was performed using Velvet (2). The resulting chromosomal contigs were oriented and assembled into one circular scaffold based on a physical map generated from optical mapping of HindIII-digested DNA fragments (OpGen). The sequence gaps were closed by primer walking and Sanger sequencing. In addition to the chromosome, we found one plasmid in the initial assembly and confirmed its circularity by PCR.

The procedure for genome annotation was largely based on that described in one of our previous studies (3). Briefly, the protein-coding genes were predicted using Prodigal (4) and annotated according to the orthologous gene in the *Escherichia coli* K-12 MG1655 genome (5), the KEGG Automatic Annotation System (KAAS) tool (6) provided by the Kyoto Encyclopedia of Genes and Genomes (KEGG) database (7, 8), and the BLASTp (9, 10) hits in the NCBI nonredundant protein database (11). The gene names and product descriptions were manually curated to incorporate information from these different sources. The rRNA and tRNA genes were predicted and annotated using RNAmmer (12) and tRNAscan-SE (13), respectively.

The genome of *S. marcescens* WW4 consists of one circular chromosome (5,241,455 bp; 59.6% G+C content) and one circular plasmid, pSmWW4 (3,248 bp; 47.8% G+C content). The first version of annotation includes 4,809 protein-coding genes (4,806 on the chromosome and three on the plasmid), 79 tRNA genes, and 22 rRNA genes, in seven operons. Our preliminary examination of the gene content identified one complete *pig* gene cluster for the biosynthesis of prodigiosin (i.e., a red pigment with antibiotic activities), which shares the same gene organization with *S. marcescens* ATCC 274 (14). Additionally, several putative bacteriocin genes exist in the *S. marcescens* WW4 genome, which are likely to be responsible for the intergeneric inhibition phenotype of this bacterium (1).

### Nucleotide sequence accession numbers.

The complete genome sequence of *S. marcescens* WW4 has been included in the GenBank Whole-Genome Shotgun (WGS) database under accession no. CP003959 (chromosome) and CP003960 (plasmid pSmWW4).
